# Altered expression levels of *IDH2* are involved in the development of colon cancer

**DOI:** 10.3892/etm.2012.676

**Published:** 2012-08-20

**Authors:** QIANG LV, SHENYANG XING, ZHENXIAO LI, JIANHUA LI, PENGTAO GONG, XIAOFANG XU, LE CHANG, XIAOXIA JIN, FENG GAO, WEI LI, GUOCAI ZHANG, JU YANG, XICHEN ZHANG

**Affiliations:** 1 College of Animal Science and Veterinary Medicine, Jilin University, Changchun 130062;; 2 The Affiliated Hospital of Jilin Medical College, Jilin 132000, P.R. China

**Keywords:** *IDH2*, expression level, colon carcinoma

## Abstract

*IDH2* encodes a mitochondrial metabolic enzyme that converts isocitrate to α-ketoglutarate (α-KG) by reducing nicotinamide adenine dinucleotide phosphate (NADP^+^) to NADPH and participates in the citric acid cycle for energy production. Notably, this gene has been shown to be critical for cell proliferation. The abnormal expression of *IDH2* has been reported in several types of cancer, and mutations in *IDH2* have been identified in gliomas and acute myelogenous leukemia. The overexpression of *IDH2* has been reported in endometrial, prostate and testicular cancer as well as in Kashin-Beck disease. In this study, we observed that *IDH2* expression was significantly downregulated in early phase but was upregulated in advanced phase colon carcinoma compared to peritumoral tissues. In addition, we demonstrated that the growth of a colon carcinoma cell line was inhibited by IDH2-siRNA and increased following transfection with an IDH2-overexpressing plasmid. These results indicate that *IDH2* may play a unique role in the development of colon carcinoma.

## Introduction

Colorectal cancer (CRC) is one of the most common malignances in the world and ranks as the fifth most common cancer type and the fourth most common cause of cancer-related mortalities in China (1). The incidence of CRC has increased rapidly in recent years, particularly in economically developed coastal cities (2,3). Although previous studies have greatly improved our understanding of the mechanisms of CRC, the etiology and pathogenesis remain unclear. Previous studies have shown that the dysfunction of energy metabolism pathways is involved in the occurrence and development of CRC (4–6). For example, the expression of fatty acid synthase, which is a key anabolic enzyme of biosynthesis of fatty acids, plays an important role in the growth and pathogenesis of colon carcinoma (7). Uncoupling protein-2, a mitochondrial membrane protein, with an expression level that is significantly higher in colon cancer tissue than in adjacent non-cancerous tissue, may play a role in the development of colon cancer by negatively regulating the production of reactive oxygen species (8–12). Mutations of three enzymes in the tricarboxylic acid (TCA) cycle, or Krebs cycle, which is a vital process involved in energy metabolic pathways, have been reported in several types of cancers. Two of those enzymes are *bona fide* tumor suppressors: succinate dehydrogenase, which has mutations associated with paraganglioma and pheochromocytoma, and fumarase, which has mutations associated with renal carcinoma and leiomyomatosis (13,14). The third enzyme, isocitrate dehydrogenase (IDH), has also been recently shown to be involved in gliomas and acute myeloid leukemia (AML). These mutations may predispose cells to neoplasia either by activating oncogenes or inactivating tumor-suppressor genes.

IDH is a key player in the TCA cycle and catalyzes the oxidative decarboxylation of isocitrate to produce α-ketoglutarate (α-KG). The activity of IDH is dependent on nicotinamide adenine dinucleotide phosphate (NADP^+^), and the biochemical reaction catalyzed by IDH leads to the production of NADPH, which plays an important role in the cellular control of oxidative damage (15). Intact IDH activity is necessary for cellular protection from oxidative stress, and the deregulation of its functions may be involved in the development of certain types of cancers, including glial tumors (16), AML and nervous system tumors (17). The human genome has five *IDH* genes that encode three distinct IDH enzymes with activities that are dependent on either NADP^+^, such as *IDH1* and *IDH2*, or nicotinamide adenine dinucleotide (NAD^+^), such as *IDH3*. The other genes in the family include *IDH5* and *IDH6*.

The *IDH2* gene is located on chromosome 15q26.1 and contributes to the conversion of isocitrate to α-KG in the citric acid cycle for energy production in the mitochondria and is critical for proliferating cells. *IDH2* and *IDH1* mutations occur frequently in certain types of World Health Organization grade 2–4 gliomas and in AML cases with a normal karyotype (18). To date, the mutations observed in the *IDH2* gene all occur in the Arg140 and Arg172 codons. *IDH2* mutations may result in a gain-of-function ability to catalyze the conversion of α-KG to 2-hydroxyglutarate, which is an onco-metabolite and may be used as a screening and diagnostic marker. In addition, this type of mutation may contribute to tumorigenesis and provide a protective mechanism in cancers that possess *IDH2* mutations (19).

To date, all reported *IDH2* and *IDH1* mutations are heterozygous with cells retaining one wild-type copy of the relevant *IDH1* or *IDH2* allele. In addition, no reports have shown concomitant *IDH1* and *IDH2* mutations (19). Although *IDH1* mutations have been reported in colon cancer (20,21), no *IDH2* mutations in this cancer subtype have been identified to date.

Despite the widely accepted view of the functional importance of mutations in cancer, the influence of protein expression levels is also important in tumorigenesis of CRC, including the expression of *IDH2*. Thus far, *IDH2* has been shown to be overexpressed in endometrial (22), prostate and testicular cancers (23) as well as Kashin-Beck disease (24). In addition, it has been observed that siRNA knockdown of *IDH2* significantly reduces the proliferative capacity of 293T cells expressing wild-type *IDH2* (19). Shin *et al* (25), hypothesized that *IDH2* may play an important role in regulating apoptosis, since the number of apoptotic cells was markedly increased in *IDH2* siRNA-transfected HeLa cells compared to control cells after exposure to heat shock.

In this study, we observed that *IDH2* gene expression was significantly downregulated in early stage (*in situ* carcinoma) but upregulated in advanced stage (infiltrating carcinoma) CRC compared to peritumor tissue by cDNA microarray and may play a role in tumorigenesis of the disease. To test this hypothesis, we used overexpression and siRNA-mediated knockdown of *IDH2* to investigate the role of the gene in the growth of colonic carcinoma HCT-8 cells using an MTT assay. In addition, we assayed the alteration of IDH activity by IDH2 in transfected cells to explore the influence of the enzyme on the proliferation of the HCT-8 colon carcinoma cell line. Our results indicated that *IDH2* may play an important role in the development of colon cancer.

## Materials and methods

### Patients

Five phase IIb T2N1M0 (*in situ* carcinoma) and 5 phase IVa T4N2M1 (infiltrating carcinoma) colon carcinoma samples based on the TNM classification of malignant tumors as well as adjacent peritumor tissues were obtained from patients who had surgery without previous radiation or chemotherapy at the Affiliated Clinical Hospital of Jilin University (Jilin, China). The patients had an age range of 35–58 years (mean 44). The tissue samples were snap-frozen and stored in liquid nitrogen for further RNA processing. Patient informed consent was obtained and ethics approval was granted from The Affiliated Hospital of Jilin Medical College.

### cDNA microarray

A cDNA microarray chip (22K Human Genome Array Chip; CapitalBio, Co., Ltd., Beijing, China) was used for the tumor or peritumor tissue samples in this study. The cDNA microarray chip was constructed by CapitalBio Co., Ltd. as previously described by our laboratory (26). Briefly, the total RNA was extracted using TRIzol reagent and further purified using a NucleoSpin^®^ RNA clean-up kit (Takara, Otsu, Shiga, Japan). The RNA samples were then reverse-transcribed to cDNA, which was marked by fluorescence (Amplification labeling kit; GE Healthcare, Buckinghamshire, UK) and amplified. After hybridization, washing and chip scanning (Jingxin LuxScan 10KA dual-channel laser scanner; CapitalBio, Co., Ltd.), the results were analyzed by significance analysis of microarray (SAM) software (version 3.0; Stanford University, Stanford, CA, USA) to determine differentially expressed genes.

### IDH2 RNAi and vector construction

The siRNA duplexes for *IDH2* were synthesized using the PGPU6/GFP/Neo siRNA expression vector by Gene Pharma (Shanghai, China). The siRNA construct that targeted the *IDH2* sequence (5′-GGCGTTTCAAGGACATCTTCC-3′) was synthesized and the PGPU6/GFP/Neo siRNA expression vector plasmid was used as a negative control.

Human *IDH2* full-length cDNA was generated by RT-PCR from total RNA isolated from HCT-8 cells. The primers used in this study are listed in Table I. The purified PCR products were then subcloned directly into the multicloning site (MCS) of the pMD-18T vector (Takara). The gene was then digested by *Eco*RI and *Hin*dIII restriction enzymes and subcloned into the MCS of a pEGFP-C1 vector. The constructed gene was sequenced to confirm the orientation and reading frame of the gene.

### Cell culture and transfection

HCT-8 cells were cultured in RPMI-1640 (Invitrogen-Gibco, Grand Island, NY, USA) supplemented with 10% heat-inactivated fetal bovine serum (Haoyang Biological Manufacture Co. Ltd., Tianjin, China), 2.30 mg/ml NaHCO_3_, 2.38 mg/ml HEPES (pH 7.2), 4 mM glutamine, 50 U/ml penicillin and 50 mg/ml streptomycin at 37°C in a humidified atmosphere containing 5% CO_2_.

For the transfection experiments, HCT-8 cells grown in 6-well plates at 70–80% confluency were covered with 2 ml of complete medium. Concurrently, 7 μg plasmid and 4 μl FuGENE^®^ HD transfection reagent (Roche, Mannheim, Germany) were diluted in 500 μl RPMI-1640 without serum. Following incubation for 5 min. The diluted DNA plasmid and FuGENE HD transfection reagent were mixed together and incubated at room temperature for an additional 20 min. This combination was then added to the wells containing cells. Six hours later, the medium was replaced with complete medium and the cells were cultured for an additional 18 h. The transfected cells were then used for subsequent experiments.

### Quantitative real-time RT-PCR

This experiment was conducted as previously described by our laboratory (26). Briefly, the total RNA of transfected HCT-8 cells was extracted using the Simply P Total RNA Extraction kit (BioFlux, Hangzhou, China). After quantification by an ultraviolet spectrophotometer, 2 μg of total RNA was reverse-transcribed to cDNA. The cDNA (2 μl) was then used for quantitative PCR amplification as previously described by our laboratory (26). The primers are listed in Table II. Gene expression levels were measured in at least 3 independent experiments.

### Western blot analysis

Transfected HCT-8 cells were lysed in lysis buffer (50 mmol/l Tris-HCl pH 8.0; 1% Triton X-100; 100 μg/ml PMSF; and 0.150 mmol/l NaCl) and the protein concentration was determined using a BCA protein assay kit (Pierce, Rockford, IL, USA). Equal amounts of protein (20 μg) were then boiled in 5X sample buffer and resolved by 12% SDS-PAGE. After being transferred and blocked with 5% non-fat milk, the membranes were probed with an anti-human IDH2 antibody (Abcam, Cambridge, MA, USA) followed by an anti-mouse secondary antibody conjugated to horseradish peroxidase (Santa Cruz Biotechnology, Inc., Santa Cruz, CA, USA). The protein bands were then visualized with a diaminobenzidine (DAB) kit (Bioer Technology, Inc., Hangzhou, China). An anti-β-actin antibody (Wuhan Boster Biological Technology, Ltd., Wuhan, China) was used to assess equal protein loading in all lanes.

### IDH activity assays

To assess the effects of IDH2 protein on enzymatic activity, an IDH activity kit (Genmed Scientifics Inc., Arlington, MA, USA) was used to assess IDH2-transfected HCT-8 cells. The cells were collected 24 h after transfection, subjected to centrifugation at 1000 × g for 10 min at 4°C, washed once with cold phosphate-buffered saline (PBS), lysed in the lysis buffer used for western blot analysis sample preparation, and then centrifuged at 12,000 × g for an additional 30 min. The IDH activity of the supernatants was measured using the manufacturer’s instructions. The activity of IDH was measured in at least 3 independent experiments at 25°C by spectrophotometry (UV-2802H; Unico, China).

### Cell proliferation assay (MTT)

To explore the possible effects of *IDH2* on cell growth, an MTT assay was used to measure cell proliferation in transfected HCT-8 cells. The cells were collected 24h after transfection, diluted in medium and then seeded into 96-cell culture wells (4,000 cells/well). At the indicated times, 20 μl MTT (5 μg/μl in PBS) reagent was added to each well, and the cells were cultured for an additional 4 h. After the incubation, 150 μl DMSO was added to each well and mixed for 10 min by continuous agitation. The absorbance values were then measured at a wavelength of 490 nm by an enzyme-linked immunosorbent assay (ELISA).

### Statistical analysis

The statistical analysis program SPSS v.13.0 was used for all experiments (IBM, USA). The results were expressed as mean ± standard error (SEM) and the statistical analysis was carried out using two-way analysis of variance (ANOVA). The significant overall differences were detected by ANOVA and the differences between treatments were compared using a Student’s two-tailed unpaired t-test. P<0.05 was considered to indicate a statistically significant result.

## Results

### IDH2 expression is significantly increased in colon cancer

**Figure 1:**
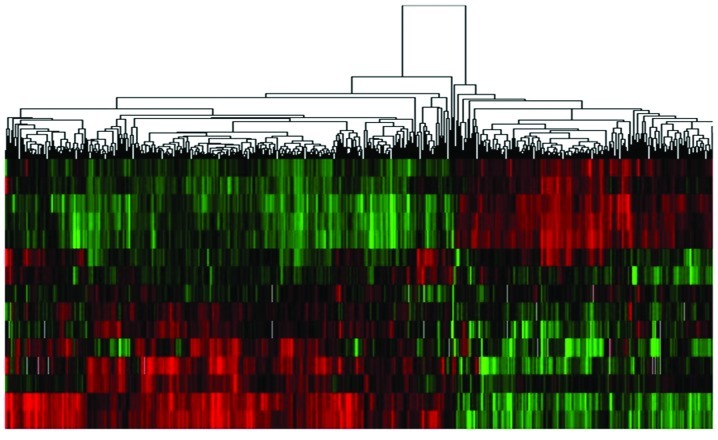
Gene expression in colon carcinoma samples compared to adjacent peritumor samples as determined by microarray. The red signals represent higher expression of genes and the green signals represent lower expression of genes.

The microarray data obtained from colon cancer samples of 5 patients identified 3,622 differentially expressed genes, including 1,757 upregulated and 1,865 downregulated genes in infiltrating carcinoma compared with the peritumor tissues. In addition, 4,055 differentially expressed genes, including 2,058 upregulated and 1,997 downregulated genes, were identified from *in situ* carcinoma samples compared to peritumor tissues (Fig. 1). Within this analysis, we observed that *IDH2* expression was significantly downregulated in *in situ* carcinoma and upregulated in infiltrating carcinoma compared with peritumor tissues (Table III).

### The expression of IDH2 in HCT-8 cells transfected with IDH2 siRNA or overexpression plasmid

**Figure 2:**
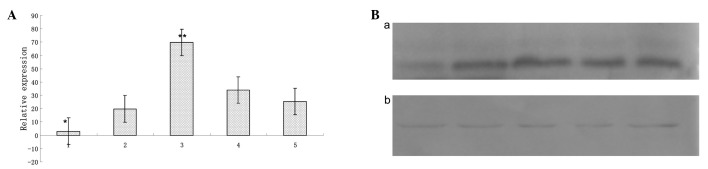
*IDH2* expression levels in HCT-8 cells. (A) Expression of *IDH2* mRNA was altered in HCT-8 cells transfected with *IDH2* siRNA or the overexpression plasmid as detected by quantitative real-time RT-PCR. The experiment was performed in triplicate and repeated twice. Error bars represent the standard deviation, with ^*^P<0.05 and ^**^P<0.01 by the Student’s t-test. 1, HCT-8 cells transfected with PGPU6/GFP/Neo-*IDH2* plasmid; 2, HCT-8 cells transfected with PGPU6/ GFP/Neo vector plasmid; 3, HCT-8 cells transfected with pEGFP-C1-*IDH2* plasmid; 4, HCT-8 cells transfected with pEGFP-C1 vector plasmid; 5, HCT-8 cells without plasmid. (B) Western blot analysis confirmed a marked reduction in IDH2 protein levels in cells transfected with *IDH2* siRNA and markedly increased expression in cells transfected with the *IDH2* expression vector. The cells transfected with empty vector and the untreated cells were used as controls. (a) the *IDH2* group; (b) the β-actin group.

The expression of *IDH2* mRNA in HCT-8 cells transfected with *IDH2* siRNA or the overexpression plasmid was detected by quantitative real-time RT-PCR. The expression level of *IDH2* mRNA in cells transfected with the siRNA plasmid was significantly lower than that in cells transfected with control siRNA or non-transfected cells (Fig. 2A; P<0.05). The cells transfected with the *IDH2* plasmid had significantly higher levels of IDH2 expression than control cells (Fig. 2A; P<0.01). In addition, western blot analysis confirmed a marked reduction in IDH2 protein levels in cells transfected with IDH2 siRNA and markedly increased expression in cells transfected with the *IDH2* expression vector (Fig. 2B).

### The influence of IDH2 expression on IDH activity

**Figure 3:**
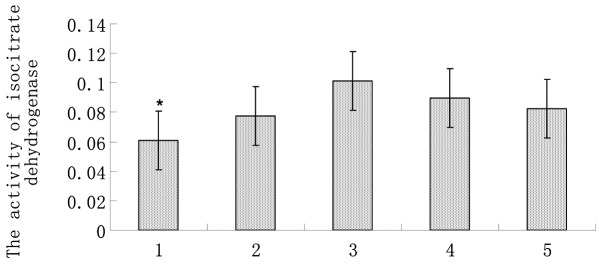
The activity of isocitrate dehydrogenase (IDH) in HCT-8 cells. The IDH activity in cells transfected with IDH-siRNA was lower than that in the control cells. The cells transfected with the empty vector and untreated cells were used as controls. The experiment was performed in triplicate and repeated twice. Error bars represent the standard deviation, and ^*^P<0.05 by a Student’s t-test. 1, HCT-8 cells transfected with the PGPU6/GFP/Neo-*IDH2* plasmid; 2, HCT-8 cells transfected with the PGPU6/GFP/Neo vector plasmid; 3, HCT-8 cells transfected with the pEGFP-C1-*IDH2* plasmid; 4, HCT-8 cells transfected with the pEGFP-C1 vector plasmid; 5, HCT-8 cells without plasmid.

We then assessed the effect of IDH2 expression on IDH activity in HCT-8 cells. The results demonstrated that the IDH activity in cells transfected with *IDH2* siRNA was lower than cells transfected with vector control or non-transfected cells. In addition, the IDH activity in cells transfected with the *IDH2* overexpression plasmid was higher than that in control cells, but the difference was not significant (Fig. 3).

### The influence of the expression levels of IDH2 on cell growth

**Figure 4:**
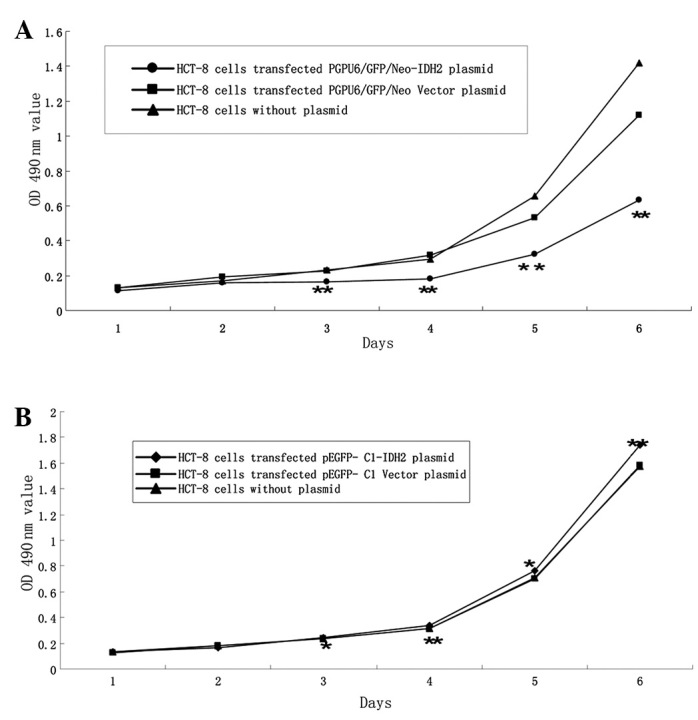
The growth of HCT-8 cell is affected by the expression of IDH2. An MTT assay was used to assess HCT-8 cell growth after reduction or overexpression of IDH2. (A) Cell growth was significantly decreased by transient transfection of PGPU6/GFP/Neo-*IDH2* starting on day 3 post-transfection compared to controls. The cells transfected with the PGPU6/GFP/Neo empty vector and untreated cells were used as controls. (B) Cell growth increased after transient transfection of the pEGFP-C1-*IDH2* vector by Day 3 post-transfection compared to controls. The cells transfected with pEGFP-C1 empty vector and untreated cells were used as controls. The experiment was performed in triplicate and repeated twice. Error bars represent the standard deviation, and ^*^P<0.05 and ^**^P<0.01 was statistically significant as determined by the Student’s t-test.

To investigate whether the expression level of the *IDH2* gene contributes to colon cancer development, the repression and overexpression of *IDH2* was examined in HCT-8 cells using the MTT assay to assess the effects on cell growth. Repression of *IDH2* significantly inhibited cell growth (Fig. 4A; P<0.05), while the overexpression of *IDH2* promoted cell growth (Fig. 4B). These data indicated that *IDH2* plays an important role in controlling HCT-8 cell growth and may also be involved in the development of colon cancer.

## Discussion

The dysregulation of enzymes in the TCA cycle is a common phenomenon in cancer. IDH is a key player in the TCA cycle and catalyzes the oxidative decarboxylation of isocitrate to α-KG while reducing NADP^+^ to NADPH and contributing to the NADPH production required for nucleotide and lipid biosynthesis during cell growth (27). The dysfunction of IDH through mutation or alteration in expression level has been observed in numerous types of cancers, which indicates that IDH may have a unique role in tumorigenesis. Although *IDH2* has been demonstrated to be upregulated in several types of human cancers, no studies have shown alteration of *IDH2* in colon cancer. Our data demonstrate that *IDH2* was significantly downregulated in early stage (*in situ* carcinoma) and upregulated in advanced stage (infiltrating carcinoma) colon cancer compared to peritumor tissue (Fig. 1). Therefore, *IDH2* may be an important factor in the development of colon cancer. To test this hypothesis, we reduced IDH2 expression by transfecting *IDH2*-siRNA or overexpressed the gene by transiently transfecting the *IDH2* plasmid to investigate the role of *IDH2* in the growth of HCT-8 colon carcinoma cells. We observed that cell growth was inhibited by siRNA transfection and promoted by transfection of the overexpression plasmid. These data demonstrate that the expression level of *IDH2* may be critical for the proliferation of HCT-8 colonic carcinoma cells.

To determine whether IDH activity influences the proliferation of colonic carcinoma cells, we assayed the alteration of IDH activity in cells transfected with *IDH2* siRNA or the overexpression plasmid. The results showed that IDH activity was lower in cells transfected with the siRNA plasmid, but not significantly higher in cells transfected with the overexpression plasmid compared to cells transfected with the control vector or non-transfected cells. These data indicated that both IDH activity and alteration of IDH2 expression affects the proliferation of HCT-8 cells.

There are several possible reasons why *IDH2* may be critical for cell proliferation. First, since IDH2 converts isocitrate to α-KG through the reduction of NADP^+^ to NADPH, IDH2 may affect cell proliferation through alterations in NADP levels. NADP, which includes NADP^+^ and NADPH, is known to be critical for anti-oxidation and reductive biosyn-thesis (28–30), however, a rapidly growing body of evidence suggests that NAD and NADP play critical roles in cell death as well. The main roles of NADP link cell survival with various biological properties, including energy metabolism (a critical factor that determines cell death), mitochondrial function, oxidative stress (a key factor in cell death), anti-oxidation, and gene expression (including caspase-dependent endonuclease GFF40, NAD^+^-dependent sirtuins, and the NAD- or NADP-dependent enzymes PARP-1, GAPDH and AIF) (31,32). Second, IDH2 may affect cell proliferation through cytosolic NADP^+^-dependent isocitrate dehydrogenase (IDPc), which controls the redox balance and provides a cellular defense against oxidative damage. It has been shown that NIH3T3 cells with low levels of IDPc become more sensitive to cell death when exposed to singlet oxygen. However, cells with highly overexpressed IDPc exhibit enhanced resistance against singlet oxygen compared to control cells (33). These data indicate that IDPc plays an important anti-protective role in singlet oxygen-induced apoptosis, possibly by acting as an antioxidant enzyme.

The *IDH2* gene is critical for cell proliferation. Although mutations and abnormal expression of *IDH2* have been identified in several types of cancers, mutations in the *IDH2* gene have not been described in colon cancer. In this study, we observed that *IDH2* is downregulated in *in situ* colonic carcinoma and upregulated in infiltrating colonic carcinoma compared with normal tissues by cDNA microarray. This indicates that *IDH2* may have a role in the development of colon cancer. To identify whether the expression level of *IDH2* affects the proliferation of colonic carcinoma cells, we assayed the growth of the HCT-8 colonic carcinoma cell line after transfection with IDH2-siRNA or the overexpression plasmid. These data indicated that the knockdown of *IDH2* caused a reduction in cell growth, while overexpression promoted cell growth *in vitro*. Based on these findings, we hypothesize that the abnormal expression level of *IDH2* contributes to the development of colon carcinoma, which is in agreement with previous studies (19). *IDH2* may therefore act as an oncogene, however further studies are required to confirm this hypothesis.

## Figures and Tables

**Table I:** Primers for full-length IDH2 cloning.

Gene	Primer sequences	Length of fragment
*IDH2*	Forward: 5′-GCGAAGCTTCTCCCGCCCTGCTCGTTCGCTCT-3′	1,585 bp
	Reverse: 5′-GCGGAATTCTAAACGCACTGCTCCTGCCTCACG-3′	

The underlined sequences indicate *Eco*RI and *Hin*dIII restriction sites, respectively.

**Table II:** Primers and probes for the quantitative real-time RT-PCR assay.

Gene	Primer sequences	Length of fragment
*IDH2*	Forward: 5′-CAAAAACATCCCACGCCTAGTC-3′	103 bp
	Reverse: 5′-TGAAACACCGTCTGGCCC -3′	
	Probe: 5′-CCATGGCGACCAGTACAAGGCCA-3′	

**Table III:** Characteristic value of the *IDH2* gene in different stages of colon carcinoma samples compared to adjacent peritumor tissues.

Gene	Stage of carcinoma	Score (d)	Numerator (r)	Denominator (s+s0)	Fold change
*IDH2*	IIb T2N1M0 (*in situ* carcinoma)	−2.33702	−1.42193	0.60844	0.31073
	IVa T4N2M1 (infiltrating carcinoma)	+0.18456	+0.13961	0.75647	0.99932

The numerator (r) and denominator (s+s0) are the results in the date process. The score is the ratio of numerator and denominator. ‘+’ and ‘−’ of the score indicates upregulation and downregulation of the gene, respectively.
